# Extended family thalassemia screening as a feasible alternative method to be implemented in identifying carriers in West Java, Indonesia

**DOI:** 10.1007/s12687-021-00565-w

**Published:** 2021-11-16

**Authors:** Susi Susanah, Nur Melani Sari, Delita Prihatni, Puspasari Sinaga, Jessica Oktavianus Trisaputra, Lulu Eva Rakhmilla, Yunia Sribudiani

**Affiliations:** 1 Department of Child Health, Hematology-Oncology Division, Dr. Hasan Sadikin General Hospital/Faculty of Medicine, Universitas Padjadjaran, Bandung, 40161 Indonesia; 2 Departement of Clinical Pathology, Dr. Hasan Sadikin General Hospital/Faculty of Medicine, Universitas Padjadjaran, Bandung, 40161 Indonesia; 3 Faculty of Medicine, Universitas Padjadjaran, Bandung, 40161 Indonesia; 4 Department of Public Health, Epidemiology and Biostatistic Division, Faculty of Medicine, Universitas Padjadjaran, Bandung, 40161 Indonesia; 5 Department of Biomedical Sciences, Biochemistry and Molecular Biology Divison, Faculty of Medicine, Universitas Padjadjaran, Bandung, 40161 Indonesia; 6 Study Center of Medical Genetics, Faculty of Medicine, Universitas Padjadjaran, Bandung, 40161 Indonesia

**Keywords:** Thalassemia carrier, Extended family, Screening, Feasible

## Abstract

The thalassemia screening program in Indonesia mostly conducted sporadically. Ideal prospective screening is still limited. This study aimed to compare thalassemia screening methods using the extended family approach with and without a history of severe thalassemia and the feasibility of implementing extended family screening method. A case control study was conducted in Dr. Hasan Sadikin General Hospital Bandung with 3 generations of extended families. Data were collected from 150 subjects of 8 extended families with severe thalassemia as an index case entry and 151 subjects of 12 families with no history of thalassemia. All subjects were examined for Hb, MCV, MCH, and peripheral blood smear (PBS) as initial laboratory examinations. Subjects with MCV < 80 fL, MCH < 27 pg, and suggestive findings on PBS continued hemoglobin analysis. Carrier status was determined by definition. All subjects consented to undergo screening and voluntarily participated. The proportion of thalassemia carriers and the participation rate between the 2 groups were compared. Sixty-four of 150 (42.7%) and 16 of 151 (10.6%) carriers were identified in both the case and control group (*p* < 0.001). The participation rate was 42–88 vs. 23–100% (*p* = 0.244). The mean age was 31.9 ± 21.2 vs. 31.1 ± 20.8 years (*p* = 0.782). The median family size was 28.5 vs. 20 subjects per family (*p* = 0.245). The types of identified thalassemia carrier in both groups consisted of β-thalassemia, β-thalassemia/HbE, suspected α-thalassemia, and β-thalassemia Hb variant. All carriers continued the counseling process. The extended family method seems feasible to be implemented for thalassemia screening in West Java, Indonesia.

## Introduction

Thalassemia is one of the most common genetic blood disorders and is inherited mostly in an autosomal recessive manner following Mendel’s laws. Genetically, it is characterized by a mutation in the α- or β-globin gene causing decreased or no production of the α- or β-globin chain in red blood cells. This condition induces hemolytic anemia, which later develops into chronic anemia. Patients suffering from this disease require lifelong blood transfusion and other medications to achieve a longer life expectancy and better quality of life (Cappellini et al. 2014; Payandeh et al. 2014). Mutation of the β-globin gene (HBB, NM_000518) is common in the population around the thalassemia belt region, including Indonesia (Fucharoen and Winichagoon 2011). Globally, around 7% of pregnant woman carry thalassemia or hemobinopathy and over 1% of couples are at risk of having children with a thalassemia disease (Modell and Darlison 2008), while in Indonesia, the Eijkman National Molecular Institute reported that the prevalence of β-thalassemia carriers is approximately 3–10% (Sofro 1995), whereas the prevalence of α-thalassemia carriers is approximately 2.6–11% (Setianingsih et al. 2003). Assuming that approximately 5% of the Indonesian population are thalassemia carriers, it is predicted that approximately 2,500 new thalassemia major cases will occur per year (Wahidiyat et al. 2020). The burden of affected families is immense, and the cost of treatment means that thalassemia is a catastrophic disease that produces not only medical problems but also psychosocial and financial problems (Ministry of Health Indonesia 2018; Wahidiyat et al. 2020). To date, this disease is ranked fifth among chronic diseases and continues to burden the national health insurance program (National Health Insurance of Indonesia 2019). Without the implementation of an effective prevention program, it seems that thalassemia will continue to be a major healthcare problem.

Thalassemia is a preventable disease, and it is well known that prevention efforts could reduce the incidence of this disease. Prevention of thalassemia can be accomplished by hindering marriage between thalassemia carriers to prevent the birth of a child with thalassemia major. Hence, the major effort is to identify thalassemia carriers. Worldwide screening and prevention programs can be implemented prospectively or retrospectively. The prospective screening method applies thorough screening to the general population to identify thalassemia carriers before and at childbearing age before they have an affected child and target screening, which is restricted to a particular population group, such as couples preparing to marry, before conception or in early pregnancy. Prospective carrier identification is more appropriate for populations with a high frequency of thalassemia. However, retrospective screening is conducted when couples already have an affected child. Practically, family member approach for severe thalassemia patients is the index case, which adopts either immediate or extended family screening or both (Ansari et al. 2012). Retrospective screening is often performed in populations with a low frequency of thalassemia or at the initiation of a prevention program in a high-frequency population (Angastiniotis et al. 2013). Studies in many countries have found extended family screening with some modifications successful in identifying thalassemia carriers (Ahmed et al. 2002; Ansari et al. 2012; Baig et al. 2008; Gorakshakar and Colah 2009; Jain et al. 2012). Countries have found that thalassemia prevention programs are more beneficial than allowing uncontrolled progress of the disease (Cousens et al. 2010; Pauzy et al. 2018; Viprakasit et al. 2013).

To date, thalassemia screening programs in Indonesia have mostly been conducted sporadically by researchers and the results are used to represent the national prevalence of thalassemia carriers. While the numbers of thalassemia major or transfusion-dependent thalassemia (TDT) patients mostly based on reports from parents’ associations registry. A national screening program will be implemented soon. Due to economic constraints, the screening program was identified as cost-effective and as a feasible method for nationwide applicability. This study aimed to compare thalassemia screening methods using extended families approach with and without a history of severe thalassemia and the feasibility of implementing extended family screening method.

## Methods

A case control study was conducted in Dr. Hasan Sadikin General Hospital, Bandung, Indonesia, from February to September 2020, involving 3 generations of extended families. Ethical approval was obtained from the Ethical Committee of Medical Faculty of Universitas Padjadjaran/Dr. Hasan Sadikin General Hospital, Bandung, Indonesia. This study was conducted according to the principles of the Declaration of Helsinki, and the period of recruitment was 8 months.

Eight families with children with severe thalassemia as index case entries and twelve control families with no history of thalassemia were offered testing to identify carriers. The criteria for selection were voluntary participation and the availability of many family members. A meeting was arranged with three generations of each family to educate and explain the importance of testing to identify other carriers. After meeting and counseling, written consent from all subjects was obtained voluntarily to participate and undergo screening. When family representatives agreed, a three-generation pedigree (index case, siblings, parents, parent’s sibling and families, grandfather and grandmother) was drawn up, and arrangements were made for testing family members. Upon obtaining written informed consent, approximately 5.0 mL of venous blood was collected from each participant in an ethylenediaminetetraacetic acid (EDTA)-anticoagulated blood sample tube. The results were given to the carriers themselves (or to the parents when a carrier was less than 18 years old).

All subjects were examined for Hb, mean corpuscular volume (MCV), and mean corpuscular hemoglobin (MCH) simultaneously using an automated hematology analyzer (Sysmex XN 1000) and peripheral blood smear (PBS) as initial laboratory examinations. Screening for thalassemia carriers was based on MCV, MCH, and hemoglobin electrophoresis. A MCV value less than 80 fL and/or a MCH value of less than 27 pg (for children according to age) and a suggestive finding on PBS were used as cutoff levels to initially identify participants as potential thalassemia carriers. These are the widely recommended RBC indices to continue to hemoglobin analysis using capillary electrophoresis. Sebia capillary electrophoresis was performed for thalassemia carrier detection (Giordano 2013; Stephens et al. 2012). If the HbA_2_ level was more than or equal to 3.5%, HbF and HbE level higher than normal for age, HbA_2_ level was less than 3.5%, and presence of Hb variant, it was concluded as β-thalassemia, β-thalassemia/HbE, suspected α-thalassemia, and β-thalassemia Hb variant carriers, respectively (Giordano 2013; Stephens et al. 2012). All identified carriers continued the counseling process, which was adjusted according to the age of the subjects. For subjects less than and equal to 15 years old, counseling will be delivered to parents or guardians; for those more than 15 years up to 18 years old delivered to the subject accompanied by parents or guardians; while for those more than 18 years of age, counseling was delivered directly to the subject.

### Statistical analysis

The collected data were analyzed using SPSS version 25.0. The frequency of subject characteristics and screening test results were calculated. Student’s independent *t* test or the Mann–Whitney test was used for comparisons between both groups. A *p* value of < 0.05 was considered statistically significant in all analyses.

## Results

**Fig. 1:**
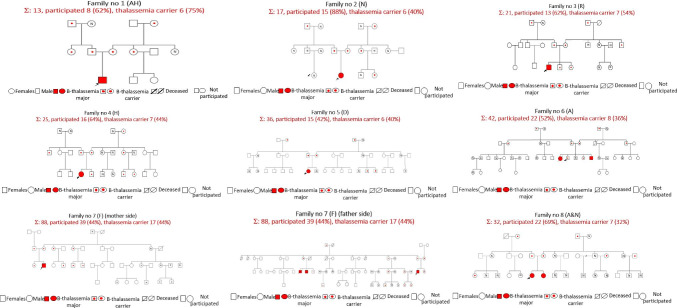
Pedigree of index case families (all). Arrow (in diagonal) indicates index case

**Fig. 2:**
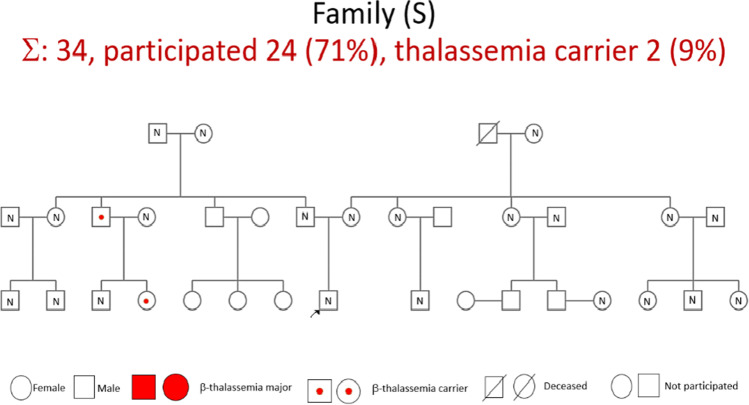
Pedigree of control family

Sixty-four of 150 (42.7%) carriers were identified in the case groups vs. 16 of 151 (10.6%) in the control groups, which was significantly different (*p* < 0.001). The mean age was 31.9 ± 21.2 (range 1–92) vs. 31.1 ± 20.8 (range 1–78) years old (*p* = 0.782), and the median family size was 28.5 (range 13–88) vs. 20 (range 13–46) subjects per family (*p* = 0.245). Clinical parameters, including Hb, MCV, and MCH, were significantly different between the case and control groups (*p* < 0.05). The mean hemoglobin A_2_ (HbA_2_) levels in carriers were 4.7 ± 1.0; 5 (1.7–6.3) in the case group vs. 2.5 ± 0.5 and 2.4 (1.8–3.3) in the control group (*p* < 0.001). The type of identified thalassemia carriers in both groups consisted of β-thalassemia carrier, β-thalassemia HbE, suspected α-thalassemia, and β-thalassemia Hb variant (53 (83%) vs. 5 (31%); 8 (13%) vs. 2 (13%); 2 (3%) vs. 9 (56%); 1 (1%) vs. 0 (0%), respectively). A participation rate of more than 40% (range 42–88%) was reported in the index case families with a mean of 60.4%, while in the control group, the participation rate was more than 20% (range 23–100%) with a mean of 60.4% (Table 1). In 8 extended families with an index case, all parents as the second generation (100%) were confirmed as having the β-thalassemia trait. In 4/8 index cases, siblings had the β-thalassemia trait: in one family, both children were severe β-thalassemia patients, one family had a normal sibling, and the remaining (2/8) index cases were only children. From the first generation of 8 extended families, it was found that either grandfathers or grandmothers from the father and mother of the index case were identified as having β-thalassemia traits. In 5/8 extended families with index cases, cousins (third generation) were identified as having β-thalassemia traits. A summary of the characteristics of the extended family with index cases of thalassemia major and with no history of thalassemia is shown in Tables 2 and 3. Figure 1 provides the pedigree of the case group, and Fig. 2 shows an example of a pedigree from the control group.

**Table 1:** Characteristics and clinical parameters of the case and control groups

Variable		Cases group 8 extended families (*n* = 150)	Control group 12 extended families (*n* = 151)	*p* value
Age, *n* (%)				
	≤ 18 years old	52 (34.7%)	50 (33.1%)	0.776
	> 18 years old	98 (65.3%)	101 (66.9%)	
Sex, *n* (%)				
	Male	71 (47.3%)	64 (42.4%)	0.388
	Female	79 (52.7%)	87 (57.6%)	
Participant rate, *n* (%)				
	Mean ± SD	60.4 ± 5.2	60.4 ± 6.4	0.244
	Median (range)	62 (42–88)	64 (23–100)	
Hb (g/dL)				
	Mean ± SD	13.2 ± 1.8	13.7 ± 1.9	0.025
	Median (range)	13.2 (5.1–17.7)	13.6 (8.3–18.1)	
MCV (fL)				
	Mean ± SD	75.4 ± 10.4	83.6 ± 6.8	< 0.001
	Median (range)	78.8 (55.4–95.7)	84.4 (59–104.1)	
MCH (pg)				
	Mean ± SD	24.9 ± 4.3	27.8 ± 2.8	< 0.001
	Median (range)	26.1 (14.4–31.8)	28.3 (15.9–35.8)	
Peripheral blood smear, *n* (%)				
	Microcytic hypochrome	80 (53.3%)	25 (16.6%)	< 0.001
	Normal	70 (46.7%)	126 (83.4%)	
HbA_2_				
	Mean ± SD	4.7 ± 1.0	2.5 ± 0.5	< 0.001
	Median (range)	5 (1.7–6.3)	2.4 (1.8–3.3)	
HbF				
	Mean ± SD	0.2 ± 0.5	0.3 ± 0.6	0.354
	Median (range)	0 (0–3.4)	0 (0–1.8)	
Type of thalassemia carrier, *n* (%)				
	β-Thalassemia trait, *n* (%)	53 (83%)	5(31%)	≤ 0.001
	β-Thalassemia/HbE, *n* (%)	8 (13%)	2(13%)	
	Susp α-thalassemia, *n* (%)	2 (3%)	9(56%)	
	β-Thalassemia Hb variant (Hb-N-Baltimore), *n* (%)	1 (1%)	0(0%)	

*Notes: (1) SD is standard deviation. (2) Hb is hemoglobin. (3) MCV is mean corpuscular volume. (4) MCH is mean corpuscular hemoglobin. (5) HbA_2_ is hemoglobin A_2_. (6) HbF is hemoglobin F

**Table 2:** Summary of characteristics of the extended family with index cases of thalassemia major

Family no	Total family members	Participated, *n* (%)	Thalassemia trait, *n* (%)	Parents**	Siblings***		Grandfather* Grandmother* Grandfather* Grandmother				Cousins*** with thalassemia trait
				Thalassemia trait (%)	Total, *n*	Thalassemia trait (%)	Father		Mother		
Index case***											
1 (AH)	13	8 (62%)	6 (75%)	100	0	-	Thalassemia trait	Normal	N/A	Thalassemia trait	N/A
2 (N)	17	15 (88%)	6 (40%)	100	0	-	Thalassemia trait	Normal	Normal	Thalassemia trait	+
3 (R)	21	13 (62%)	7 (54%)	100	2	100%	Thalassemia trait	Normal	Thalassemia trait	(†)	2/4 normal, 2/4 N/A
4 (H)	25	16 (64%)	7 (44%)	100	1	100%	Thalassemia trait	Normal	Normal	Thalassemia trait	+
5 (D)	36	15 (42%)	6 (40%)	100	1	-	N/A	Thalassemia trait	Thalassemia trait	Normal	+
6 (A)	42	22 (52%)	8 (36%)	100	4	50%	Thalassemia trait	Normal	Thalassemia trait	Normal	6/13 normal, 7/13 N/A
7 (F)	88	39 (44%)	17 (44%)	100	1	100%	Thalassemia trait	(†)	Normal	(†)	+
8 (A&N)	32	22 (69%)	7 (32%)	100	0	-	(†)	Thalassemia trait	Thalassemia trait	Normal	+

*N/A* did not participate; (†) died; + presence

*first generation; **second generation; ***third generation

**Table 3:** Summary of characteristics of the extended family with no history of thalassemia

Family no	Total family members	Participated, *n* (%)	Thalassemia trait, *n* (%)	Parents**	Siblings***		Grandfather* Grandmother* Grandfather* Grandmother*				Cousins***
				Thalassemia trait (%)	Total, *n*	Thalassemia trait (%)	Father		Mother		with thalassemia trait
Index case***											
1 (L)	41	13 (32%)	0 (0%)	0	4	-	(†)	Normal	N/A	N/A	2/4 normal, 2/4 N/A
2 (B)	13	13 (100%)	0 (0%)	0	0	-	Normal	Normal	Normal	Normal	1/1 normal
3 (S)	34	24 (71%)	2 (8%)	50	0	-	Thalassemia trait	Normal	(†)	Normal	+
4 (AL)	21	14 (67%)	1 (7%)	50	0	-	Thalassemia trait	Normal	Normal	Normal	2/6 normal, 4/6 N/A
5 (AW)	13	10 (77%)	2 (20%)	50	0	-	N/A	N/A	Thalassemia trait	Normal	2/2 normal
6 (F)	13	11 (85%)	0 (0%)	0	2	-	N/A	Normal	N/A	Normal	1/1 normal
7 (M)	22	5 (23%)	2 (40%)	50	0	-	N/A	N/A	N/A	Thalassemia trait	5/5 N/A
8 (TU)	18	11 (61%)	2 (18%)	50	2	-	Normal	Normal	Thalassemia trait	Normal	2/4 normal, 2/4 N/A
9 (R)	15	10 (67%)	1 (10%)	50	2	-	Thalassemia trait	Normal	(†)	Normal	2/2 normal
10 (AD)	19	10 (53%)	1 (10%)	50	1	-	(†)	Normal	Thalassemia trait	Normal	3/3 N/A
11 (G)	23	11 (48%)	2 (18%)	50	2	-	(†)	(†)	Thalassemia trait	Normal	+
12 (FM)	46	19 (41%)	3 (16%)	50	7	14	N/A	N/A	Thalassemia trait	Normal	+

*N/A* did not participate; (†) died; + presence

*first generation; **second generation; ***third generation

## Discussion

There are several possible strategies for thalassemia screening depending on the factors to be considered, such as the frequency of the disease; heterogeneity of the genetic defects; available resources; and social, cultural, and religious factors. However, the available technical facilities, infrastructure, and financial resources affect both the strategy and the choice of methods for carrier identification (Angastiniotis et al. 2013).

Many countries have conducted their own strategies based on nationwide capacities. Screening can be categorized as mandatory or voluntary. Despite the WHO recommendation that no compulsory genetic testing should be conducted, some countries, including Iran, Saudi Arabia, and Palestinian territories, have laws in place making hemoglobinopathy screening mandatory for all couples before obtaining approval to marry. In Cyprus, couples waiting to get married are required by the church to be screened and counseled. In other countries, including Sardinia, Greece, Guangdong Province of China, and England, hemoglobinopathy screening programs are offered on a voluntary basis. The success of thalassemia prevention programs has been demonstrated in Cyprus, Greece, and the UK (Angastiniotis et al. 2013; Cousens et al. 2010).

Retrospective or inductive screening (also known as cascade screening or extended family testing) involves the testing of relatives of identified carriers and/or patients and is a powerful means of improving the efficiency of carrier identification (Angastiniotis et al. 2013). Some countries have used a combined approach regarding resources, such as Sardinia and Malaysia, which generally use prospective strategies; however, in some states, screening is conducted retrospectively.

In Sardinia, for instance, such a policy has led to the detection of 90% of expected at-risk couples through tests on only 15% of the adult population (Cousens et al. 2010). Pauzy et al. (2018) reported the result of a thalassemia screening in Sabah, an East Malaysian state located in Borneo that has many indigenous ethnic groups. Of 645 blood samples obtained from all over Sabah, 94% of the total sample came from voluntary screening, and 30% (193/645) were identified as thalassemia and/or hemoglobinopathy carriers, most of whom had β-thalassemia traits (23%). This is much higher than the estimated rate of β-thalassemia carriers in the Malaysian population of 3–5% (Ibrahim 2009).

Ideally, as a country with a high frequency of thalassemia, Indonesia should implement mass screening, which can be mandatory or voluntary (Angastiniotis et al. 2013; Maskoen et al. 2019; Modell and Darlison 2008). Several thalassemia screening studies in Indonesia have demonstrated various results. Sofro AS (1995) reported that there was a 3–10% β-thalassemia carrier rate in Indonesia. Lanni et al. (2004) also reported a rate of thalassemia carriers in Jogjakarta of 1.5–36% HbE/β-thalassemia carriers and 2.6–11% α-thalassemia carriers.

The present study was conducted in Bandung, West Java Province, Indonesia**,** which has a high number of patients with thalassemia major. To date, West Java Province, with a population of 50 million, which is almost one-fifth of the Indonesian population (approximately 268 million), is the province with the highest incidence of thalassemia major; almost 40% (4,199/10,555) of cases originate from this region (Working Group of Hematology Oncology Indonesian Pediatric Society 2020). Several isolated studies and thalassemia screening programs have reported quite different results. The latest report from Maskoen et al. (2019), in a cross-sectional study on family gathering for Thalassemia Day in Bandung, West Java, reported that 13.7% of family members of thalassemia major patients were identified as HbE/β-thalassemia carriers. Most of the studies or screenings were provided for families who had an index case, retrospectively.

Most thalassemia carriers in the case group were β-thalassemia which is in line with the most common of thalassemia type in Indonesia (Ministry of Health Indonesia 2018). Meanwhile in the control group, the majority was identified as suspected α-thalassemia and further examination is needed to confirm the precise diagnosis. However, at least 7/151 = 4.6% had confirmed as thalassemia carrier.

This study demonstrated that 42.7% of thalassemia carriers were identified from 8 extended families of the case index, much more than in the control group, which identified 10.6% thalassemia carriers from 12 extended families without a history of thalassemia. The incidence of thalassemia carriers in the control group (10.6%) was in line with the incidence of thalassemia carriers in previous study reported by Sofro AS (1995), Lanni et al. (2004), and other sporadic/sentinel screenings of the general population in some regions of Indonesia (Husna et al. 2017; Rujito et al. 2014; Susanti et al. 2017). It seems the control group who were originated from families without thalassemia history can be considered to represent the general population who screen prospectively.

As we expected, we found the prevalence 4 times higher carrier thalassemia in the extended family of cases group. It means, for retrospective screening, practically extended family approach is more efficient. This cascade or extended family screening has been applied in previous studies in some countries. In previous studies in Pakistan, Ahmed S. et al. in 2002 identified 31% β-thalassemia carriers in the extended family of an index case as a case group, while there were no thalassemia carriers identified in the control group; Baig SM et al. in 2009 identified 44.4% β-thalassemia carriers; Ansari SH et al. in 2012 identified 62.2% of siblings identified as β-thalassemia carriers as opposed to 5–8% carriers in the general population; and Majeed T. et al. in 2013 found 61% of different types of thalassemia disorders, including 51.9% for the β-thalassemia trait. In a study in India in 2009, Gorakshakar AC et al. reported a 21.9% rate of β-thalassemia carriers in an extended family of thalassemia major patients; this result was 5–6 times higher than the incidence of thalassemia carriers in the general population. Jain et al. conducted a study with “risk group screening” in 2012 (West Bengal, India) and found that 55.8% were identified as β-thalassemia carriers, followed by 15.7% as carriers of HbE/thalassemia.

In this study, we investigated families in three generations and draw it in a three-generation pedigree. We also elaborated their family history through in-depth history taking. Assessment of family history is useful to detect increased risks for diseases that have modifiable risk factors or preventable exposures (Brock et al. 2010). When a relatively common disease is caused by an inherited mutation in a single gene, family history assessment may lead to early diagnosis and more aggressive management (Wattendorf and Hadley 2005). Furthermore, extended family in three generations will assess risk factors for inherited or multifactorial disorders that may be amenable to risk screening, genetic testing, prenatal diagnosis, and disease prevention or management. It can reveal information (such as early onset disease, close relationships between individuals with disease, multiple affected family members, suspicious a kind of disease history, or diseases with known genetic basis) and provides a pictorial representation of diseases within a family and is the most efficient way to assess hereditary influences on disease (Wattendorf and Hadley 2005).

Practically, it can be initiated by determining an index case and making their own pedigree, then tracing all of the family members with their baseline data, including their addresses, and mapping them to the closest primary healthcare facilities that have thalassemia screening facilities. This process can be facilitated by using a simple (Android) application, so it can be implemented by many family members.

In taking the family history, it is important to note the ethnicity of the four grandparents. Ethnic background can be a risk factor for significant inherited conditions that may be screened for carrier status and subsequently tested (for instance, thalassemias and hemoglobinopathies). Clinicians should be familiar with specific populations within their region of practice that may have carrier risks for inherited disorders. It is also necessary to include the evaluation of the possibility of consanguinity preference. Consanguinity not only increases the risk of multifactorial conditions, but also has the potential (an estimated tenfold increased risk) to expose rare autosomal recessive conditions in offspring through shared inheritance of gene mutations (Modell and Darr 2002; Modell and Darlison 2008). In Pakistan, a higher incidence of thalassemia and hemoglobinopathies was identified in families with cultural preferences for consanguineous marriage (Ahmed et al. 2002; Ansari et al. 2012; Baig et al. 2008; Majeed et al. 2013). In this study, based on thorough interview, there are no family members who had history of consanguineous marriage. However, in some areas in West Java Province and also in some regions in Indonesia, this culture does exist.

The extended family screening method will be able to trace the second-generation profile and thus is better able to focus on family members of productive age, including the siblings of thalassemia major patients, cousins, couples who plan to marry, couples who plan to have children, and pregnant women. Early identification of thalassemia carriers will provide time and consideration to select potential partners for those who are unmarried and antenatal screening for couples at risk (Angastiniotis et al. 2013; Gorakshakar and Colah 2009). Genetic counseling by a certified counselor should be conducted both before and after screening.

In this study, all parents of the case index were thalassemia carriers, and one grandfather or grandmother from the father’s and mother’s side of the index case was identified as a thalassemia carrier. It was found that thalassemia carriers occurred since the previous first generation, and thalassemia carriers or thalassemia major was traced in the next generation if marriage between thalassemia carriers was not avoided. Carriers pass the mutation to their offspring in an autosomal recessive manner; if they meet and marry another thalassemia carrier, they will be at risk of giving birth to either a new thalassemia carrier or a child with thalassemia major (Fucharoen and Winichagoon 2011). Most of the index cases had siblings and cousins who were identified as thalassemia carriers and were recommended to continue genetic counseling. Therefore, once an index case is identified, it is an important marker to conduct screening for the extended family (Ahmed et al. 2002; Ansari et al. 2012).

This study found that the participation rate was quite high in both the case and control groups (42–88% vs. 23–100%, respectively) and was not significantly different. The comparable participation rate between the two groups indicated that with thorough and intensive education, the extended family screening method was feasible to be implemented. This result also suggested that thalassemia screening and counseling were acceptable in extended families of severe β-thalassemia patients in Indonesia.

Thalassemia is a disease that is still unfamiliar to society. Social stigma about thalassemia also makes families tend to conceal the illness. Community education and socialization about thalassemia should continue from secondary high school to university and should be provided in the general population to couples and pregnant women (Rakhmilla et al. 2018). Thalassemia prevention programs by socialization in the population can use social media, including health promotion programs such as education. Health promotion is a process to empower members of society to be independent with the aim of maintaining and improving health (Angastiniotis et al. 2013; Khorasani et al. 2008; Singh et al. 2019). Screening can be targeted to different age groups with genetic counseling adjusted according to the age of the individual or the target group being screened (Angastiniotis et al. 2013). This study showed that regardless of the screening method, education to the public which will make them aware and understand of this disease with the importance of its prevention is one of the important keys in implementing this program beside fully government support.

The success of thalassemia screening programs could ultimately prevent new cases of newborns with severe thalassemia. Thalassemia screening in the extended family which is feasible to be implemented also efficient, we identified many more carriers than population-based screening. Furthermore, from our experience, this screening method is well accepted by family members regardless of their thalassemia carrier status. This study emphasizes that thalassemia screening in the extended family is a good candidate alternative screening method for developing countries with a high prevalence of thalassemia carriers but limited resources to initiate screening programs, such as the province of West Java, Indonesia, either as a single or in combination with restricted to a particular population group screening while waiting for the availability of resources to carry out an ideal population screening.

Although the results of the screening programs are comparable with those of other countries based on different methods, the feasibility of implementing screening methods in Indonesia, whose geographical, economic, social, cultural, and political milieu is varied, should be determined for each region.

## Conclusion

The extended family method that can obtain higher coverage seems feasible to be implemented as a potential alternative of thalassemia screening method in West Java, Indonesia. Practically, screening is focused on siblings and cousins before and at childbearing age of individuals with thalassemia major as index cases. For Indonesia’s national prevention program, it is suggested to implement a combined approach regarding resources and budgets, using a restricted prospective strategy and combining it with retrospective ones such as extended family screening.

## Data Availability

The datasets generated during and/or analyzed during the current study are available from the corresponding author on reasonable request.
